# The social and environmental factors impacting the motivation of adolescents for weight control, why and how? A qualitative study

**DOI:** 10.1186/s40795-024-00822-4

**Published:** 2024-01-30

**Authors:** Lida Shams, Fatemeh Shafiei, Zahra Sadremomtaz

**Affiliations:** 1 Department of Health Policy and Management, School Of Public Health and Safety, Shahid Beheshti University of Medical Sciences, Tehran, Iran; 2 Department of Community Nutrition, School of Nutrition Science and Food Technology, Shahid Beheshti University of Medical Sciences, Tehran, Iran; 3 Department of Community Health Education, Virtual School of Medical and Management, Shahid Beheshti University of Medical Sciences, Tehran, Iran

**Keywords:** Adolescents, Teenager, Environmental Factors, Social Factors, Obesity, Motivation, Weight loss

## Abstract

**Introduction:**

Overweight and obesity are common problems among teenagers regardless of ethnicity, race, and socio-economic status. Therefore, this study aims to explore the social and environmental factors impacting adolescents motivation for weight control in Gilan province, Iran.

**Methodology:**

Following a qualitative design, a content analysis approach was used to analyze the data. A total of 79 interviews were conducted with Adolescents (*n* = 23), Friends and Peers (*n* = 15), Parents (*n* = 12), Managers (*n* = 16), and Health care providers (*n* = 13), regarding adolescents obesity during 2019. MAXQDA V.10 software was used for our analysis.

**Findings:**

The main categories of environmental and social factors affecting adolescents motivation for weight control were external factors (the relative success of weight control intervention programs, the lack of environmental and social support, and the lack of family support for teenagers) that each one had some subcategories, and internal factors (competence, relatedness, and autonomy).

**Conclusion:**

This study demonstrated the necessity of identifying environmental and social factors that are effective in reducing adolescents’ motivation for weight loss. These factors are so influential that teenagers can’t overcome them without receiving support from their environment and the government health-related policies. So, it seems that we need integrated multisectoral approaches and we suggest that health policymakers develop practical policies to control adolescents obesity by focusing on factors that have been mentioned in this study.

## Introduction

Obesity is an important challenge of the twenty-first century and has a significant prevalence among children and adolescents in both developed and developing societies [1, 2]. WHO reported that the global prevalence of obesity almost tripled from 1975 to 2016. Besides, this rising trend is more significant among children and adolescents aged 5–19 (from 4% in 1975 to 18% in 2016) [3], and according to estimates, it is expected to be continued in the future years [2]. In Iran, as a developing country, the NIMS-II (second National Integrated Micronutrient Survey) was conducted in 2012 and reported that zone 1 (including Gilan and Mazandaran provinces) has the second adolescents obesity prevalence (8.4%) in the country [4]. Then in 2015, the results of CASPIAN-V (Childhood and Adolescence Surveillance and Prevention of Adult Non-Communicable Disease) study of 14,118 subjects aged 7 to 18 years of 30 provinces in Iran, showed that prevalence of overweight and obesity is 9.4% and 11.4%, respectively [5]. Also, a pooled analysis reported more than 20% increasement in prevalence of overweight in Iranian adolescents from 1975 to 2016 [2]. More recently, Koch project (a national plan for weight and obesity control of students) in Iran reported that Gilanian students ranked the first in terms of obesity (18.9%) and third in terms of overweight (23.2%) [6].

Childhood obesity is related to considerable potencial consequenses including medical (fatty liver disease, sleep apnea, Type 2 diabetes, asthma, hepatic steatosis, cardiovascular disease, high cholesterol, cholelithiasis, glucose intolerance and insulin resistance, skin conditions, menstrual abnormalities, impaired balance, and orthopedic problems), socio-emotional (stigma, low social acceptance, discrimination, social marginalization, low self esteem, low self confidence, negative body image), and academic consequences (low school performance, missing school) [7].

The main cause of obesity is the interaction of environmental and cultural factors [8]. Recent evidence indicated that these factors are manifested in people's lifestyles, leading to health-related behaviors such as diet type and activity level [6, 9, 10]. So, main focus of health system currently is on changing individual behavior. However, it seems that regarding to multifactorial etiology of obesity and importance of its structural barriers, this approach is not appropriate enough. Therefore, it should be paid more attention to variables like health governance issues, and the lack of coherent strategies in the food and health systems [10, 11].

One of the individual level factors is motivation. Each person needs motivation to continue living, survival, activity and even change [12]. There are two types of motivation, internal and external. Internal motivation is the result of personal reinforcements and external motivation is the result of society-related reinforcements such as social, environmental and cultural factors. And finally, motivation and desire are determinants of our health-related behaviors [13, 14].

Unfortunately, our teenagers almost live in environments that lead them to unhealthy diet and lower physical activity resulting overweight and obesity. Nutrition transition, easy access, cheap prices, and attractive advertisements of unhealthy foods alongside with sedentary entertainment are main reasons of these risk factors [9]. Therefore, it seems necessary to consider these factors during critical initial periods of life including before and during pregnancy, infancy and early childhood and adolescent ages [9].

The opinions of the target community are an essential component of designing effective patterns to obesity prevention and control [12]. So, studying the views of different stakeholders, in order to investigate the roots of obesity problem and to provide a suitable strategy for resolving it, can lead to provision of appropriate and integrated approaches [15]. Therefore, due to high prevalence of adolescence obesity in Gilan province and importance of social and environmental factors, the purpose of the current study is to show why and how these factors affect motivation of adolescents for weight control.

## Methods

This study was performed on adolescents aged 10 to 19 in Gilan province. Participants were selected using purposive sampling while emphasizing selecting participants from various socio-economic groups, urban and rural areas, and both boys and girls. The adolescents were selected using the integrated public health information system known as SIB. The inclusion criteria were being overweight or obese (using the WHO growth curves: overweight as sex‑specific BMI for age of 85th–95th, and obesity as sex‑specific BMI for > 95th (https://www.who.int/toolkits/child-growth-standards/standards/body-mass-index-for-age-bmi-for-age) and willingness to participate. Adolescents who had mobility disorders, diabetes, hypertension, cardiovascular diseases and hormonal disorders like thyroid malfunction were not included in the study. The criteria for selecting managers were having work experience in cultural, sports, educational, and health fields. Peers, parents, and health care providers who directly or indirectly dealt with adolescents were also interviewed in this study. The sampling was stopped upon reaching data saturation.

Data collection was performed via semi-structured face-to-face interviews by one of the authors (Z.SM), who is a health care provider. Each interview lasted 45 to 60 min and was audio recorded accompany to field notes. Sample interview questions for adolescents include the following: What factors motivate you to control your weight? What factors have hindered the advancement of your weight control plan? One example of questions for managers, friends and peers, parents, and health workers include the following: How do environmental and social factors influence teenagers motivation for control their weight?

Qualitative approaches are appropriate to understand the social reality of behaviors and analyze different phenomena and concepts. Content analysis is one of the methods of analyzing qualitative studies, by means of which data is summarized, described and interpreted, and it is a suitable method for examining people's experiences and attitudes towards a particular subject [16]. Therefore, this qualitative content analysis through Mayring method was used in current study in order to identify the social and environmental factors affecting adolescents motivation for weight control. This method is mainly for the analysis of personal views collected through interviews and not limited to a specific background [17]. In this regard, first, typed text of each interview was transferred to MAXQDA_10_ software. Then, according to Mayring approach, at the same time with data collection, statements that were meaningful and related to the research question were coded, reviewed several times by authors and experts and some of participants, merged similar ones together until sub-categories and upper-categories were developed. This process was performed by two researchers (L.SH and Z.SM).

We used Guba and Lincoln's indices to increase the reliability of the data. For this purpose, various methods were used such as using the opinions of different groups, allocating enough time to data collection, proper communication with the participants and conducting interviews in the places chosen by them, reading manuscripts by colleagues and people participating in the study. The data coding process was done by two persons at the same time. Purposive sampling method was used to obtain diverse and experienced stakeholders based on our inclusion criteria [18, 19]. All the activities carried out, including the work steps and how to collect and analyze the data, were carefully recorded so that it is possible to review the work steps.

Written informed consent to participate was obtained from the parents or legal guardians of any participant under the age of 16,and from the participants themselves for participants who are above the age of 16 before entering the study and after a comprehensive introduction to the study protocol. Ethics approval for this study was received from the Internal Research Ethics Committee of Shahid Beheshti University of Medical Sciences (SBUMS). Noteworthy, all methods were performed in accordance with the relevant guidelines and regulations, particularly those published by the SBUMS ethics committee.

For further deatails regarding the combined criteria to report qualitative studies see thae COREQ (Consolidated Criteria for Reporting Qualitative Research) checklist [20] in Table 1.

**Table 1:** Combined criteria to report qualitative studies (COREQ)

No Item	Guide question/description	Answer
Domain 1: Research team and reflexivity		
Personal Characteristics		
1 Interviewers/ facilitators	Which author/s conducted the interview or focus group?	Zahra Sadremomtaz
2 Credentials	What were the researcher's credentials?	Master's student in community-based education
3 Occupation	What was their occupation at the time of the study?	Student and Instructor of Health Care Training Center
4 Gender	Was the researcher male or female?	LS: Female FS: Female ZSM: Female
5 Experience and training	What experience or training did the researcher have?	Healthcare provider and qualitative studies
6 Relationship established	Was a relationship established prior to study commencement?	NO
7 Participant knowledge of the interviewer	What did the participants know about the researcher? For example, personal goals and reasons for doing the research	Prior to the interview, the participants were informed about the background, aim and procedure of the study
8 Interviewer characteristics	What characteristics were reported about the interviewer/facilitator? For example, bias, assumptions, reasons, and interests in the research topic	The interviewees were given the required information about the educational background of the interviewer and the reason for conducting the research
Domain 2: Study design		
Theoretical framework		
9 Methodological orientations and theory	What methodological orientation was stated to underpin the study? For example, grounded theory, discourse analysis, ethnography, phenomenology and content analysis	Content analysis (Methods, Paragraph 3)
Participant selection		
10 Sampling	How were participants selected? For example, purposive, convenience, consecutive, and snowball	Purposeful sampling method (Methods, paragraph 4)
11 Method of approach	How were participants approached? For example, face‐to‐face, telephone, mail, and email	Face‐to‐face
12 Sample size	How many participants were in the study?	79 (Findings, Paragraph 1)
13 Non‐participation	How many people refused to participate or dropped out? Reasons?	No one
Setting		
14 Setting of data collection	Where was the data collected? For example,home, clinic, and workplace	The places chosen by participants
15 Presence of non‐participants	Was anyone else present besides the participants and researchers?	No
16 Description of sample	What are the important characteristics of the sample? For example, demographic data and date	Demographic data, job, work experiences (Table 2)
Data collection		
17 Interview guide	Were questions, prompts, guides provided by the authors? Was it pilot tested?	Yes
18 Repeat interviews	Were repeat interviews carried out? If yes, how many?	NO
19 Audio/visual recording	Did the research use audio or visual recording to collect the data?	Audio recording
20 Field notes	Were field notes made during and/or after the interview or focus group?	Yes
21 Duration	What was the duration of the interviews or focus group?	45–60 min
22 Data saturation	Was data saturation discussed?	Yes
23 Transcripts returned	Were transcripts returned to participants for comment and/or correction?	Yes, to some of them
Domain 3: Analysis and findings		
Data analysis		
24 Number of data coders	How many data coders coded the data?	2 of authors coded the data (LS and ZSM)
25 Description of the coding tree	Did authors provide a description of the coding tree?	Yes
26 Derivation of themes	Were themes identified in advance or derived from the data?	Derived from the data
27 Software	What software, if applicable, was used to manage the data	MAXQDA software, version 10
28 Participant checking	Did participants provide feedback on the findings?	“Reading manuscripts by colleagues and people participating in the study” was done to increase the reliability of findings
Reporting		
29 Quotations presented	Were participant quotations presented to illustrate the themes/findings? Was each quotation identified? For example, participant number	Yes
30 Data and findings Consistent	Was there consistency between the data presented and the findings?	Yes
31 Clarity of major themes	Were major themes clearly presented in the findings?	Yes
32 Clarity of minor themes	Is there a description of diverse cases or discussion of minor themes?	Yes

### Findings

The characteristics of the study participants are described in Table 2. A total of 79 interviews were conducted with five groups of participants, including: Adolescents *(A)* (*n* = 23), Friends and Peers *(F)* (*n* = 15), Parents *(P)* (*n* = 12), Managers *(M)* (*n* = 16), and Health care providers *(HP)* (*n* = 13).

**Table 2:** Characteristics of the study participants

Participants	Adolescents	Friends and peers	Parents	Health care staff	Managers
Variable					
N	23	15	13	12	16
Gender					
Female/Girl	18	9	8	12	11
Male/Boy	5	6	5	0	5
Living area					
Urban	17	11	11	16	16
Rural	6	4	2	0	0
Job					
Student	23	15	-	-	-
Unemployed	-	-	5	-	-
Employee	-	-	4	12^a^	16^b^
Laborer	-	-	3	-	-
Farmer	-	-	1	-	-
Age (year)	10–17	12–17	28–60	45–53	35–60
Work experiences (year)	-	-	-	8–29	10–28

^a^(2 Health paraprofessional, 5 Healthcare provider, 1 Mental health expert, 1 Environmental health expert, 2 Adolescent and school health expert, 1 Dietitian)

^b^(5 Provincial staffs of education department, 3 Provincial staffs of MoHME (Ministry of Health and Medical Education), 3 Provincial members of the municipal council, 5 Provincial staffs of Sports and Youth department)

Health related behaviors such as diet and physical activity have an important role in morbidity and mortality around the world. So, any changes in such behaviors can have significant effects on community health and understanding factors affecting these behaviors is essential for future health interventions and policies [21]. Although there have been interventions to change the behavior of adolescents towards obesity, these interventions have not been significantly effective. As a result, adolescents have failed to do healthy behaviors. In this regard, it seems lack of supportive external and internal factors has led to decrease motivation needed to change behavior and maintain it [22]. In this regard, the factors that impact adolescents motivation for weight control are presented in Table 3 and discussed here:

**Table 3:** The factors that affected motivation of adolescents for weight control

Main category	Upper category	Subcategory
**External Factors**	Relative success of weight control interventions programs	▪ Weak inter-sectoral coordination with education system and other stakeholders ▪ Weakness in the proper implementation of the community-based nutrition program ▪ Failure to implementation of unhealthy food advertisement policy ▪ High price of healthy food
	Lack of environmental and social support	▪ Culture of residence place ▪ Promote the use of mobile-based social media
	Lack of family support	▪ Dedicating little time to cooking homemade food ▪ Allocating a small amount of money for physical activity of teenagers ▪ Lack of sufficient awareness of parents ▪ Lack of healthy eating style at home
**Internal Factors**	Competence	
	Relatedness	
	Autonomy

1. External Factors

Definition of social determinants of health by WHO, is “the conditions in which people are born, grow, work, live, and age, and the set of forces and systems shaping the conditions of daily life.” [23]. In this regard, inter personal rewards or restrictions are external regulators affecting personal motivation, from least to most self-determined level, including: extrinsically regulated extrinsic motivation, epistemic extrinsic motivation, simulated extrinsic motivation, and integrated extrinsic motivation [24].

There are a lot of external factors that affect personal health, and according to obesity, we found some of them as follow:

A Relative success of weight control intervention programs:

Participants stated that intervention programs (regarding nutrition and physical activity) do not completely support adolescents weight control.

- ▪ Weak inter-sectoral coordination with education system and other stakeholders:

They stated that in order to control weight and other risk factors of chronic diseases, there is a need for coordination between the health and education networks, so it is expected that the policies will be approved and implemented according to the needs of the target community. In this regard, managers declared that:

*“The authorities are not sensitive to the obesity and short height of the students. They do not believe in doing basic work in schools for young people to improving their health. Indeed, families, schools and whole society are not coordinated in this regard, meanwhile we should increase our children health awareness to prevent future consequences of obesity. (M)”*

*“The radio and television and government organizations do not inform about the obesity and overweight consequences. They do not inform about healthy diet such as low-fat and low-sugar diets. And even obese people are used in television advertisements! (M)”*

- ▪ Weakness in the proper implementation of the community-based nutrition program:

The participants stated that the health service providers need to convey sufficient information about healthy nutrition regarding to multiple variables such as cultural, economic, and social characteristics of community to all members of the household. So, there is a need for community-based nutrition experts to examine the eating habits of this region and design appropriate approaches based on the Gilan province.

*“There is weakness in public information regarding health-related services. For example, most people do not use nutrition counseling because this service is expensive in private sectors and they don’t know that the health centers present free nutrition counseling. (M)”*

*“There are growing wrong eating habits of the people especially in children and adolescents. We don’t have safe and health places for exercise. (A)*

- ▪ Failure to implementation of unhealthy food advertisement policy:

The participants state that media advertising can significantly affect on behavior of people, especially teenagers. In this regard, proper information about the complications of obesity is very effective for teenagers now and in the future. But instead of this, we can see the misleading advertisements of unhealthy food in our media that encouraging teenagers to use these foods.

*“Radio and television advertise high-energy and fattening foods for their financial gain, while they have been expected to promote healthy diet. (HP)”*

- ▪ High price of healthy food:

Healthy foods have a high price especially after economic sanctions against Iran in recent years [25]. The participants believed that this situation jeopardized food security and prevents access to healthier food.

*“Low-calorie and healthy foods are expensive and fast foods and unhealthy foods are cheaper. So, people tend to eat cheaper, but unhealthier foods. (HP)”*

B Lack of environmental and social support:

The participants believed that all our health policies aren’t properly implemented resulting increasement of fast-food stores all around the province especially near schools, not having a safe place for exercise and the lack of suitable space in parks, neighborhoods and villages, a large number of students in classrooms.

*“If we look at their living environment, fast-food shops have sprung up everywhere like mushrooms, and this is the reason of obesity. Next, he said: Fast food shops especially near schools have increased significantly. (HP)”*

*“There are many students in each classroom and usually teaching other lessons is prior to exercise and most exercise hours will be used for teaching other subjects. Generally, exercise hasn’t importance in school, exercise hours are not enough, as well as most schools haven’t required facilities. When weather isn’t appropriate, they spend their exercise time inside the classroom, because there is no covered place for this matter. (M)”*

*“Women especially in small towns don't want to be in the public outdoor places, so they prefer limited indoor environments(M)”*

- ▪ Culture of residence place

Participants stated that teenagers are influenced by their neighborhood meanwhile they didn’t have choose it. Therefore, to prevent being judged by other, they try to behave like them. Also, neighborhood affects on goods availability in the supermarkets.

*“Choosing high-calorie foods in supermarkets with the advice of obese friends, and spending time in cyberspace instead of exercise are barriers to weight loss(A)”*

*“I was very obese, and then I decided to walk with my dad in the morning. I didn’t do it before, because I didn’t want to walk alone. As a result, now my weight is normal and I’m very glad of it. (A)”*

*“My grandmother says: You have to lose your, otherwise you will be sick in the future. (A)”*

- ▪ Promote the use of mobile-based social media:

As we know, games and entertainment are basic needs for children and adolescents to release excitement and development of mental creativity. Unfortunately, today electronic games and mobile-based entertainment are the first choice of our children and adolescents that followed by mental problems and inability to control desires, as well as laziness, physical problems, and obesity [26]. The participants stated that the use of virtual space has increased among teenagers, especially after the outbreak of the Covid-19 epidemic, and parents pay less attention to this issue.

*“Parents are satisfied that their children sit in a quiet corner and play with their phones with no extra cost. They don't pay enough attention to teenagers diet and activities (M)”*

C Lack of family support:

Teenagers declared that their parents are a good role model for them. So, it is highly important to correct the parents' behavior to make behavioral changes in their teenagers and strengthen self-regulation in them. Parents must be supportive to their children, but some parents can’t do that because of their busy schedule, low income, family problems, and problems related to neighborhood safety and cultural factors.

*“Parents are an inappropriate role model for their children because of their unhealthy behaviors. (M)”*

- ▪ Dedicating little time to cooking homemade food:

The participants stated that families have to work long hours to earn more money and don’t have enough time to prepare homemade food, so they frequently use ready to use foods that usually are unhealthy. This is a main cause of obesity in families and teenagers.

Allocating a small amount of money for physical activity of teenagers:

Teenagers stated that their parents don’t pay them to play sports. So, they chose low price and almost sedentary activities like internet-based games and entertainments.

*“Low-income families can’t afford for sports. While, their teenagers’ favorite sports and sports club tuition are usually expensive. So, they only can use the parks! (M)”*

- ▪ Lack of sufficient awareness of parents:

Family awareness is highly important in every aspect of nutrition including shopping, preparing, cooking and serving of foodstuffs.

*“If we have basic nutritional information, we could have better diets. For example, we can exchange foodstuffs with different price but same nutritional value. (M)”*

- ▪ Lack of healthy eating style at home:

Our family members play a key role in our lifestyles.

*“I have an obese friend that she has seen a dietitian and has taken a dietary plan, but after a week, she couldn't continue her plan, because her mother didn’t accompany her. For example, her mother cooked unhealthy food and didn’t follow the plan. (P)”*

2. Internal Factors:

A previously external source of motivation is internalized and reinforced through internal pressures such as guilt and anxiety, and feelings related to self-esteem [19]. People naturally have an internal tendency to activities that are not inherently interesting but are useful for them. This internalization is motivated by three basic cognitive needs: competence, relatedness, and autonomy [18].

A **Competence:** It means the need to achieve desired outcomes and to experience dominance. Some adolescents stated that being beautiful and style satisfaction is a motivation to weight loss among adolescent.

*“If I get an award, I will follow my diet. I made it my reward to wear my favorite clothes. (A)”,*

*“Some people exercise because they have a purpose, so they try to reach it, but some people don't. (A)”*

*“Due to exercise, I feel my body will become shapelier, I will become more beautiful, and I will be more satisfied with it. So, these motivate me to continue my way, but some young people are satisfied with their body and do not try to change it. (A) ”*

B **Relatedness:** It means the need to feel connected and accepted by loved ones (friends, parents, and teachers) [27]. They declared that adolescents are very influenced by their friends and peers to choose a right or wrong behavior and they like to be approved by them.

*“My friend saw me at the store, he said, I'm going to the gym, come with me. And when I saw how well he lost weight, I agreed with him. (F)”*

*“Choosing unhealthy foods in supermarkets and spending time in mobile-based activities offering by my obese friends destroys my motivations for weight loss. (F)”*

*“Our friends are very obese, believe me. Meanwhile, they can lose their weight easily by exercise and healthy diet like reducing fat consumption, and by doing this, they become queens in the minds of others. So, push yourself to exercise and consume less high-fat diet to be a healthy child today and a healthy adult in the future for happiness of your family. (A)”*

C **Autonomy:** It means individual volition and willing to organizing own experience and behavior [27]. Some teenagers in the study stated that we can change our lifestyle to lose weight and people around us can’t decide for us. Some parents believed that they can’t make decisions for their children.

*“If someone really wants to do exercise and be active, he will provide the conditions for himself in any circumstances. For example, he can go for walking around the city, and I think it is completely safe. Or he can exercise in anywhere like parking lot or anywhere. So, the main thing is personal willing. (A)”*

*“It is personal responsibility to manage his own behavior. The home environment may not be suitable for healthy lifestyle, but if the teenager himself has proper knowledge and awareness, he can definitely overcome to his inappropriate situation. I want to be fit at this age, so I must try to get information from expert ones and use it and even share this information with my family because it can improve my family's lifestyle. (H)”*

*“We can’t interfere in the decisions of teenagers because they say that their body is their own and they don’t accept others advice. (P)”*

## Discussion

This study identified the main internal and external factors involved in adolescents motivation for weight control. As far as we know, this is the first study to evaluate these factors from perspective of the different actors in Gilan province, Iran.

One of the most important type of variables influencing adolescents motivation for weight control were external factors with three upper categories: Relative success of weight control interventionprograms was one of them. In this regard, our participants declared there is weak inter-sectoral coordination with education system and other stakeholders. Similar to our study, taghizadeh et al*.* (2021) reported that the lack of cooperation and coordination between stakeholders is a major problem to tackle childhood obesity in Iran [28]. Also, we noticed that weakness in the proper implementation of the community-based nutrition program was another external barrier. In line with our study, taghizadeh et al*.* (2021) concluded that in Iran, especially in recent decade, different policy document such as IranECHO (Ending Childhood Obesity) have been approved regarding Childhood obesity prevention, but their implementation has not been completely successful because of problems like sanctions against Iran and coronavirus disease-19 (COVID-19) pandemic [28]. Failure to implementation of unhealthy food advertisement policy was another sub-category. Also, a previous research stated that prohibition of unhealthy products advertising has been a part of health policies in Iran, but they have not been completely successful especially in the fields of TV and radio advertisements [29]. Our study showed that the high price of healthy food is a reason of adolescent obesity. As well as, a cross-sectional study from USA reported similar results and emphasis on importance of healthy food affordability [30].

Next our upper category was lack of environmental and social support including culture of residence place that have been reported as determinant factors of teenagers obesity. Similarly, Ludwig et al*.* (2011) in a randomized social experiment showed that neighborhood environment such as grocery store, exercise space, proximity to health care providers and social norms for health-related behaviors is related to obesity [31]. We found that promoting the use of mobile-based social media is important, too. Also, a population-based study of Finnish twins showed that information and communication technology can be related to adolescents’ obesity [32]. Meanwhile, we can use these technologies for obesity prevention like to monitor food intake and physical activity, and to send health messages [33].

Lack of family support including dedicating little time to cooking homemade food in another our reported barrier. As well, a cross-sectional study from Japan reported that less home cooking is linked to more childhood obesity [34]. Allocating a small amount of money for physical activity of teenagers mentioned by our participants as an obstacle, too. in Iran, the priority for most families is their children admission to high-rank universities, so they prefer that their children spend most of their time and money just for study. In low-income families, children participation in sports is limited due to fiscal problems, so a qualitative study proposed to reframe school sport interventions to support all children [35]. Lack of sufficient awareness of parents was one of our noticeable findings. According to the key role of parents knowledge, attitudes, and practices, a national survey from Singapore reported that different parenting styles have a decisive role in children obesity [36]. And, we found lack of healthy eating style at home is a problem. Also, in Gilan some nutritional habits, like consumption of appetizing native side dishes, can help to increase food intake. Similarly, Kim et al. declared that parents’ food preferences and working affects eating habits of their children [37].

Other main factors were internal factors as follow: teenagers are competing in weight, style and etc. to each other, and although they are related to their surroundings people such as family and friends, but they have their own autonomy. In this regard, a qualitative study from Norway disclosed that a social network in school can involve children in physical activity through motivating them via their basic needs including competence, relatedness and autonomy according to the Self-Determination Theory (SDT) [38]. As well as, a review article reported that these motivator needs could be applied to management of children obesity [39].

## Conclusion

According to our results, the following were the main environmental and social factors affecting adolescents motivation for weight control: external factors (relative success of weight control intervention programs, lack of environmental and social support, and lack of family support for teenagers), and internal factors (competence, relatedness and autonomy). Regarding to power of these factors, we need the integrated multisectoral approaches and developing practical health policies to tackle adolescents obesity. 

## Data Availability

The datasets used or analyzed during the current study are available from the corresponding author on reasonable request.

## Abbreviation

BMI: Body mass index
SIB: Integrated health system
